# Evaluating the Efficacy of Facial Scar Treatment Techniques Using Nanofat Grafting: A Case Series

**DOI:** 10.7759/cureus.68817

**Published:** 2024-09-06

**Authors:** Mhd Anas Alnemr, Bassel Brad, Fatima Ismail Elmi, Lei Li

**Affiliations:** 1 Department of Oral and Maxillofacial Surgery, Damascus University, Damascus, SYR; 2 Department of Maxillofacial Surgery/Plastic Operations, Klinikum Oldenburg, Oldenburg, DEU

**Keywords:** atrophic scar, depressed facial scar, facial appearance, facial scar management, iprf, modified nanofat, nanofat, unfiltered nanofat

## Abstract

Patient's psychological and aesthetic quality is affected by the appearance of scars since atrophic scars might be caused by infections or inflammations that resulted from preoperative procedures. Non-surgical methods have recently been developed to increase patient satisfaction and improve the appearance of scars.

In our understanding, there has not been much published medical research assessing how well nanofat grafting techniques work in terms of treating facial scars. With a six-month follow-up after the intervention, this research intends to provide four cases where scars in the facial area were controlled utilizing these materials. Based on the cases shown, it can be seen that following the procedure, the scar's measurements were minimized, its color and texture improved, and there was no pain or itching.

Given its safety and effectiveness, autologous nanofat grafting has become one of the most important and well-liked aesthetic procedures. It has been discussed how modifying injectable nanofat using fine needles has opened up new clinical applications for rejuvenating aesthetic medicine. Additionally, it was pointed out that injectable PRF has gained attention due to its high concentration of growth factors, which help to promote healing and improve the appearance of the skin. Furthermore, it can be efficiently shared with autogenous nanofat grafts, which support and enhance the efficacy of adipose-derived stem cells (ADSCs) and improve adipocyte blood circulation.

The current use of modified nanofat in the treatment of facial scars is supported by the procedure's safety and low risk of injection-site problems. The consistent efficacy of modified-nanofat injection in the management of depression-related facial scars was validated by the outcomes of patient satisfaction surveys and physician evaluations.

## Introduction

Scarring is the outcome of wound healing and is invariably linked to problems with functionality and cosmetic impairment [1]. Moreover, in case the scar is facial, the psychological strain that goes along with it is amplified. One frequent kind of scarring that results in a depressed deformity is called depressed scarring. On the exterior of the body. It typically develops during the healing of wounds to the skin, subcutaneous tissue, and deep tissue. Skin and soft tissue infections can also be the cause. To select an appropriate therapeutic approach, depressed scars can be separated into two groups. Initially, deeper and more severe depressed scars are those that are accompanied by subcutaneous tissue adhesion. Surgery and autologous fat grafting are frequently used in these situations [2,3]. Taking into consideration that traumatic injuries and surgical incisions play a vital role in depressed facial scars. Some could suggest that one of the main features of 21st-century scar rejuvenation therapy is fat grafting in all of its forms. Considering that appealing appearances are becoming more and more important for social survival, It makes sense that reconstructive surgery and cosmetic surgery are now inextricably linked. Scar revisions, lasers, steroid injections, skin grafting, tissue expansion, chemical peels, dermabrasion, topical lotions, and ointments are among the methods mentioned for improving the cosmetic appearance of scars.

Frame et al. [4] did mention that Neuber first proposed the idea of fat grafting in 1893. It acquired more momentum in the 1980s when Illouz [5] invented liposuction, a technique for extracting fat. Coleman [6,7] presented an improvement in his method, which is now more accepted. The discovery of multipotent stem cells marked a milestone in fat grafting.

As concentration techniques are used in the creation of nanofat and stromal vascular fraction, which produce an increased number of these products, researchers are more interested in these adipose tissue-derived products for use in clinical procedures and research. Adipose-derived stem cells (ADSCs) [8-10] are one of the best sources of progenitor cells and stem cells derived from adipose tissue are nanofat [11-13]. Due to its small pockets of bioactive peptides and stem cells, the product "nanofat" is very appealing [8]. Evidence of nanofat's capacity for tissue remodeling and regeneration has been found in dermatological conditions such as wrinkles, pigmentation, scars, tiny joints, chronic wounds, and specific ligament-tendon targets [14].

Autologous fat grafting has emerged as a potentially effective treatment option for scars in numerous domains in recent years. An inventive strategy that La Rusca et al. outlined [15] was that atrophic and depigmented forehead scars could be repaired via dermal fat grafting and non-cultured epidermal melanocyte cell suspension.

Surgeons have expressed frustration due to the fact that structural fat is very useful in correcting depressed scars [16]. Even in cases when the depression is superficial and the scar is insignificant, the fat particles remain too big. However, the idea of nanofat provided a solution to this issue, which might facilitate injection into the intra- or subdermal plane. Nanofat, which is obtained by mechanically breaking down and filtering fat, is distinguished by tissue and graft particle sizes of 400-600 μm or even smaller [17]. Tenna [18] treated acne scars using nanofat and platelet-rich plasma, and she verified that this combination improved depressed scars. This study sought to investigate the safety, durability, and consequences of using nanofat injection for the treatment of depressed facial scars in patients, given the importance of facial scar modulation and the promising outcomes of nanofat in the treatment of scars.

## Case presentation

This case series report presents four cases of patients who visited the Department of Oral and Maxillofacial Surgery Hospital at the Faculty of Dentistry at Damascus University, Damascus, Syria. These four cases shared the presence of atrophic facial scars and were treated by applying nanofat graft into scar tissue.

We used the following procedure for nanofat preparation in each case.

Nanofat grafts preparation

After identifying the patient's lower abdomen as the doner site, we inject 2% lidocaine - 1/80,000 adrenalin into the patient's navel. Following that, we performed a 2 mm incision using no. 11 blade. Next, a modified Klein solution (lidocaine 800 mg/l + adrenaline 1/1,000,000) was injected; hold off for 15 minutes. Lipoaspiration using a blunt cannula was done, and negative pressure (liposuction) was applied. After microfat was harvested, microfat was carefully transferred into many sterile 15-mL tubes and positioned symmetrically within a centrifuge.

Three layers are formed from the lipoaspirates within each tube after centrifugation at 3000 rpm (or around 1200 g) for three minutes: the top oil layer, the middle pure fat layer, and the bottom fluid part. The tube's top oil layer is decanted, and the bottom fluid is easily suctioned.

Converting microfat graft to nanofat graft

**Figure 1:**
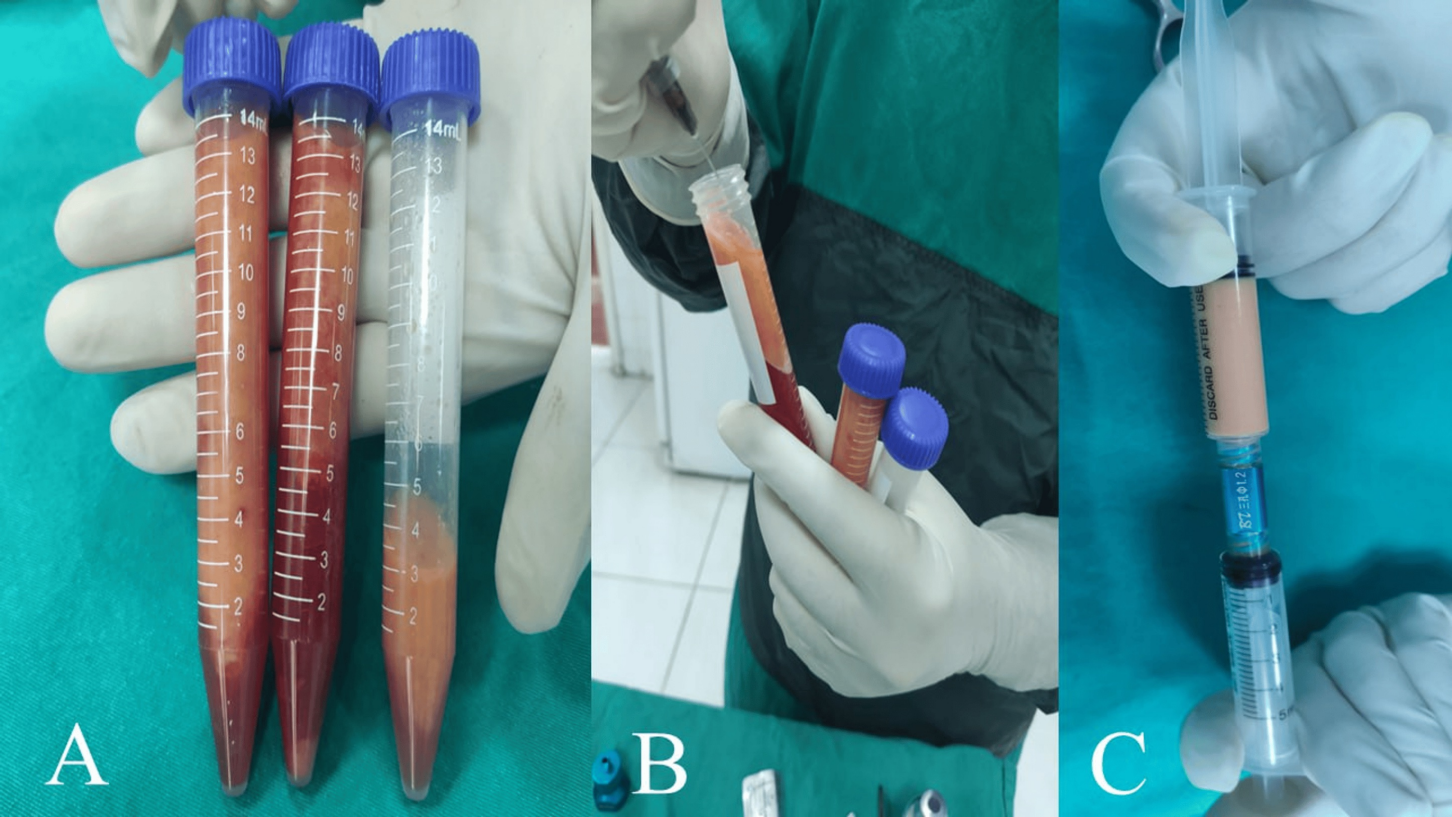
Some steps of nanofat preparation A: microfat graft, immediately after liposuction from abdomen region; B: after centrifugation of microfat graft and removing oil layer; C: converting microfat into nanofat via nanofat connector

After obtaining the pure microfat graft from the tube, it is transferred to 5 cc syringes. A mechanical emulsification process is performed and the size of the fat graft particles is minimized using a group of connectors with gradually decreasing diameters (2.0, 1.5, 1.2). After 30 alternating movements for each connector, the fat graft turns into a liquid emulsion with a whitish color. Then, a filtration process is performed using a special filter containing a mesh with ultra-fine holes to remove connective tissue debris that could cause blockage in the thin 27 Gaudge injection needles. Thus, the traditional nanofat graft is ready, according to Tonnard et al. [7]. The last filtration step of the previous protocol is deleted and the unfiltered nanofat graft is obtained. Figure 1 highlights some of the main steps in the nanofat preparation process.

Case 1

A 31-year-old woman suffered a comminuted fracture of her zygomatic and upper jaw bones in a traffic accident seven years ago. The region was deformed as a result. 3D plates printed were used to reconstruct the area. After a year of treatment, cosmetic repair of the resulting scar was done by injecting unfiltered nanofat combined with platelet-rich fibrin (PRF) into the scar tissue. The patient’s informed consent has been taken and the procedure was fully explained. Under local anesthesia with Klein solution (lidocaine 800 mg/L + adrenaline 1/1000000) into the donor site (lower abdomen) waiting 15 minutes, then microfat harvesting with a multiport 3 mm cannula with a sharp side hole of 1 mm in diameter. Microfat graft was centrifuged at 3000 rpm for three minutes, and pure fat graft was transferred to nanofat graft by using a nanofat connector (2.0-1.5-1.2), 30 passes for each connector.

I-PRF was prepared out of 20 mm collected peripheral blood and centrifuged at 700 rpm for three minutes, then mixed the unfiltered nanofat graft with I-PRF.

**Figure 2:**
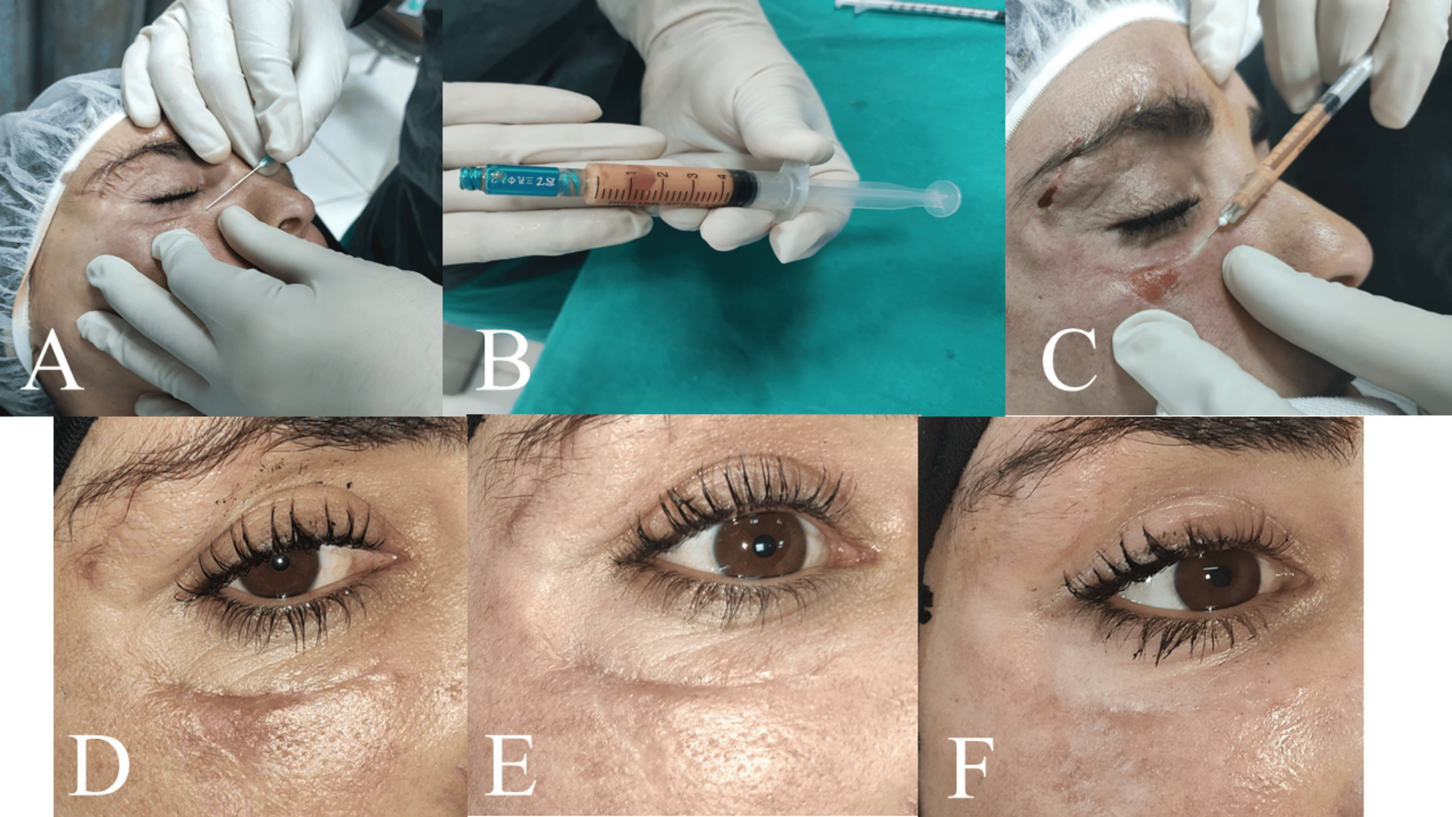
Subcision of the targeted scar (A); mixing the unfiltered nanofat graft with I-PRF (B); injecting the mixture into scar tissue; (C) atrophic scar - preoperative (D); three months postoperative (E); six months postoperative (F)

This mixture was injected into the scar tissue until the tissue started to appear yellowish after the subcision of scar tissue with a 21-gauge needle; the follow-up to this case was 3-6 months, as shown in Figure 2.

Case 2

**Figure 3:**
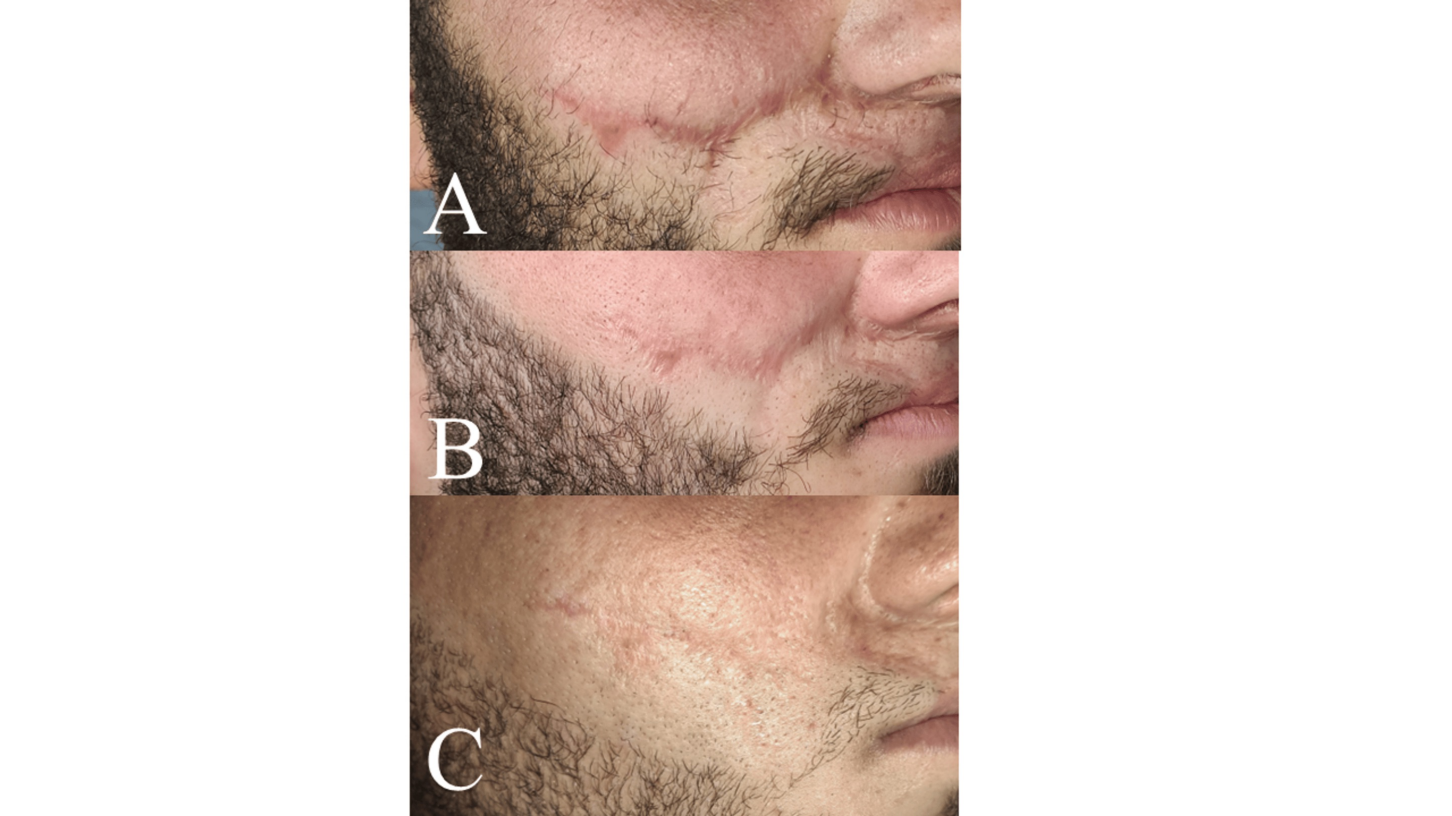
Atrophic scar - preoperative (A), three months postoperative (B), six months postoperative (C)

A 25-year-old male was involved in a traffic accident six years ago on a motorcycle, which resulted in a severe wound with lacerations and tissue loss in the upper lip. The patient refused to undergo secondary surgical correction; he tends to minimally invasive procedures. Therefore, cosmetic treatment of the formed scars was performed by intra-scar tissue injection of unfiltered nanofat and mixing them with injectable PRF with the same method described in case 1, as shown in Figure 3.

Case 3

**Figure 4:**
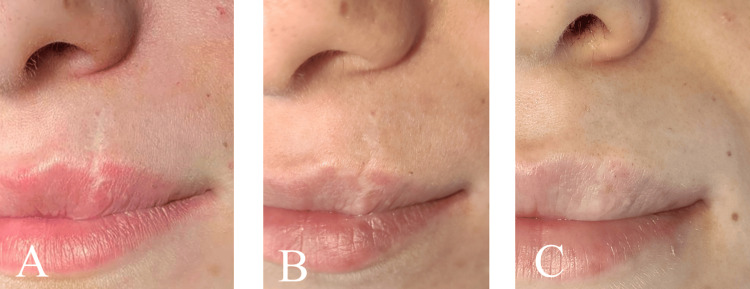
Atrophic scar - preoperative (A), three months postoperative (B), six months postoperative (C)

A 26-year-old female suffered a severe injury due to a sharp ending foreign body on the upper lip 10 years ago. The formed scar was treated by injecting conventional nanofat. After giving a full explanation of the procedure, the patient has given their informed consent. After applying local anesthetic and injecting a Klein solution (800 mg/L of lidocaine and 1/1000000 of adrenaline) into the donor site (lower abdomen), waiting for 15 minutes, and using a multiport 3 mm cannula with a sharp side hole of 1 mm in diameter, microfat was harvested. After centrifuging the microfat graft for three minutes at 3000 rpm, the pure fat graft was moved to the nanofat graft using the nanofat connectors (2.0-1.5-1.2), 30 passes for each connector. The fat graft changed into an emulsion; after this emulsification process, the fat graft was filtered and then injected into the scar tissue, as shown in Figure 4.

Case 4

A 24-year-old female patient suffered many cuts from sharp objects 10 years ago. The patient had aesthetic treatment, consisting of standard nanofat graft injections, to address the multiple created scars. Cosmetic treatment of the formed scars was performed with intra-scar tissue injections of conventional nanofat with the same method described in case 3, as shown inFigure 5.

**Figure 5:**
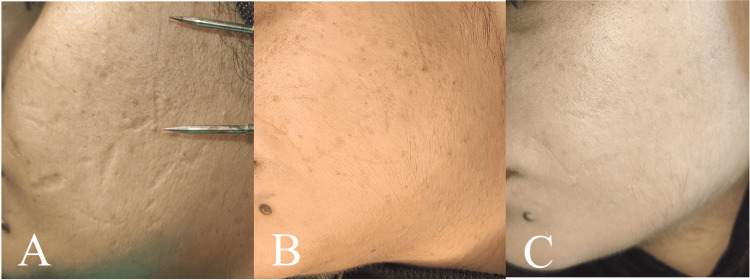
Atrophic scar - preoperative (A), three months postoperative (B), six months postoperative (C)

## Discussion

There is an ongoing rise in facial injuries, bruises, accidents, and various surgical interventions, and these cannot heal without leaving traces, such as scars, which are undesirable from an aesthetic perspective and negatively affect the patient's social connections and psychological state. The ideal scar should resemble a natural skin tone and not have any noticeable deformities [18]. The scarring that follows tissue injury is not the same as tissue regeneration, which is the true restoration of the healthy structure of the skin in all of its components and only develops in the embryo during the first two-thirds of pregnancy [19]. It is important to note that there are more than 100 million patients worldwide who suffer from these scars.

This is what led medical professionals and academics to develop a wide range of therapeutic approaches that vary depending on the kind of scar. The most prevalent type of scars on the face are atrophic scars [18]. Atrophic scars are more challenging to treat than hypertrophic scars [20]. Combination treatments are necessary to achieve the best possible clinical results for scars because non-invasive and conservative scar treatments often fail to produce the desired results for patients and physicians [21]. Current and emerging trends in scar treatment also point to the use of cell-based regenerative treatments for atrophic scars that are anticipated to permanently, highly, and efficiently yield the maximum therapeutic advantage.

Given that autologous fat is widely accessible, easy to obtain, inexpensive, and compatible with the body, it has gained approval and popularity in the field of reconstructive and regenerative medicine. It is also a great choice for facial regenerative treatments [22]. Furthermore, autologous fat has a higher concentration of stem cells than bone marrow. ADSCs have been shown in numerous studies to play a major role in wound healing, promoting regeneration, and restoring processes by promoting the development of new blood vessels, releasing growth factors, improving collagen deposition and skin elasticity, as well as tissue remodeling, thickening the dermis, and inhibiting melanin cell activity [23].

Since these nanofat grafts represent a rich source of ADSCs, it became possible to inject them into thin 27-gauge needles that fit fine areas of the face after Tonnard and his colleagues described nanofat that resulted from the process of mechanical emulsification and filtration of microfat [7].

Thus, it is imperative to guarantee the survival of cells and adipose tissue constituents, and the likelihood of cell survival rises with reduced nanograft modification. Furthermore, as Lo Furno pointed out, the unfiltered nanofat has more cells in it when the last filtration stage in the preparation of the nanofat is skipped. Denser cellular inclusions, growth factors, and ADSCs are produced from adipose tissue [24].

Hence, PRF is a second-generation blood derivative that contains high concentrations of platelets and is more suitable than PRP because it is less expensive and easier to apply clinically as it can be obtained in a single centrifugation step immediately after blood collection without the addition of any antibodies. Coagulation or stimulants, fibrin adopts a three-dimensional structure that captures concentrated platelets allows the release of growth factors and cytokines [25], and provides a scaffold for the proliferation, differentiation, and activity of ADSCs [26]. Clinically, the use of PRF in combination with auto lubricants has shown better results and an increase in the rate of doctor and patient satisfaction compared to auto lubricants alone [27].

## Conclusions

Our research indicates that, as compared to conventional nanofat injection alone, the injection of unfiltered nanofat combined with PRF into atrophic facial scars produced better cosmetic outcomes and decreased the defective appearance. After the follow-up, patients were extremely satisfied with both approaches, nevertheless.

Both approaches are highly effective, safe, and one of the most reliable minimally invasive procedures in the field of facial aesthetics. Adding injectable platelet-rich fibrin to the unfiltered nanofat graft plays a significant role in improving the clinical appearance.
